# The Meaning of Social Support in Nature-Based Services for Young Adults with Mental Health Problems

**DOI:** 10.3390/ijerph19031638

**Published:** 2022-01-31

**Authors:** Anne Mari Steigen, Bengt G. Eriksson, Ragnfrid Eline Kogstad, Daniel Bergh

**Affiliations:** 1 Department of Health and Nursing Sciences, Faculty of Social and Health Sciences, Inland Norway University of Applied Sciences, P.O. Box 400, 2418 Elverum, Norway; bengteriksson670@gmail.com (B.G.E.); ragnfrid.kogstad@inn.no (R.E.K.); 2 Faculty of Arts and Social Sciences, Karlstad University, 651 88 Karlstad, Sweden; 3 Department of Education and Special Education, The University of Gothenburg, 405 30 Gothenburg, Sweden; daniel.bergh@gu.se

**Keywords:** social support, mental health problems, nature-based services, young adults

## Abstract

In previous studies, social context and social support have been found to be important in nature-based services. However, no studies have previously focused on the meaning of different dimensions of social support in these contexts. The aim of this study is therefore to uncover dimensions of social support in relation to mental health among young adults with mental health problems participating in nature-based services in Norway. This study applies data from a survey of 93 young adults participating in nature-based services; in addition, qualitative interview data from 20 interviews are also used. The data are analysed using qualitative content analysis, descriptive statistics, and correlation analysis. The results indicate that participants in nature-based services experience emotional, esteem, informational, and instrumental support in addition to social integration and opportunities for nurturance in these services. The service leader, other participants, and the animals are important contributors to these dimensions of social support. Nature-based services may be a helpful intervention for young adults with mental health problems. The unique context of these services, including nature and animals, adds special qualities to mental health and social work practices.

## 1. Introduction

Nature-based services offer therapy or treatment interventions specifically designed, structured, and facilitated for individuals with a defined need [1]. The overall goal for all nature-based services is to provide health-promoting activities for various groups of people. Salutogenic factors, factors that might strengthen health (e.g., social support), are therefore at the core of these services.

Nature-based services are provided for people with dementia, mental health- or drug-related problems, as work training and as a pedagogical resource for a variety of participating groups. The services are often provided in a farm milieu (at or in connection to agricultural farms), and activities typically include working with animals and plants, and maintenance work [2]. Nature-based services in farm milieus are found in several European countries, including the Netherlands, the United Kingdom, and Norway [3,4,5,6]. There are various concepts used to describe these types of services. In this study, nature-based services are used; however, other common concepts describing similar services are, for example, green care and care farming [1].

Research on nature-based services for people with mental health problems has previously shown positive results with regard to participants’ mental and physical health [1,7]. The social milieu of nature-based services is considered to be one of the central mechanisms for health promotion [1,8,9,10,11]. Due to their specific challenges (e.g., mental health problems), many participants find themselves in a situation in which they tend to be socially excluded [8]. The social context of the services offers social contact, inclusion, affiliation, and support [1,3,7,8,11]. In qualitative studies, the service leaders in nature-based services are described as important by the participants [4]. Nature-based services also offer participants the opportunity to help and support one another [6]. In addition, participants in nature-based services report that the company and support of animals is very important and emphasise that it can be easier to be ‘social’ with an animal than with humans [12,13].

Numerous studies have demonstrated the positive effects of social support on mental health, coping, and quality of life, as well as on physiological health [14,15,16,17,18]. Social support and social relations might therefore be of specific importance for health promotion for participants in nature-based services with mental health problems.

A range of definitions of the concept of ‘social support’ can be found in the research literature. Cobb [19] describes social support as the individual’s experience of being surrounded with love and care, being respected and valued, and having a social network with mutual commitments. Different dimensions of social support are described, for instance emotional support, esteem support, instrumental support, informational support, and social integration. Emotional support refers to relationships that offer acceptance, intimacy, and emotional sharing. Esteem support represents the strengthening of a person’s sense of competence, for example, through positive feedback on skills or belief in coping abilities. Instrumental support refers to help with, for example, goods or services. Informational support refers to the advice or guidance one can receive from supportive people. Social integration is reflecting a person’s feeling of being part of a group that shares common interests and concerns [20,21]. Weiss [22] also emphasises that having the opportunity to provide care to others is an important dimension of social support.

Social support can further be divided into the two categories of structural and functional support. Structural support refers to a person’s degree of perceived integration into a social network, while functional support often refers to the actual support that may be provided by the social network [20,23,24,25].

Furthermore, several different scales intended to measure social support are found in the body of research. One principal distinction is between scales that attempt to measure the actual support received, and those that measure the perceived availability of support. The research suggests that perceived social support has the greatest impact on mental health [23,26].

Even though social support has been reported as being important in previous studies on nature-based services, to our knowledge, no other study has focused particularly on social support, and different dimensions of social support within these services. In addition, no research has, as far as we know, previously focused on the relation between mental health and social support, among young adults with mental health problems participating in nature-based services in farm milieus. More knowledge about social support in these services are needed to better understand how these services might be helpful for young adults with mental health problems, and what special qualities of social support these services might offer. In addition, this knowledge is important for improvement of existing services, and when establishing new ones. Knowledge about dimensions of social support and its relation to mental health in nature-based services might also have transfer value to other services for young adults with mental health problems.

The aim of this paper is to uncover dimensions of social support in relation to mental health among young adults with mental health problems, participating in nature-based services.

## 2. Materials and Methods

The data originate from two data collections—one quantitative and one qualitative—that are both parts of a larger research project about nature-based services in Norway. The participants in both data collections had left school or work, either partly or totally, primarily because of mental health-related problems, which was the inclusion criterion for participating in this study.

### 2.1. Participants in the Quantitative Sample

The quantitative data are based on a survey of young adults aged 16–30 participating in nature-based services. All ongoing nature-based services in Norway were contacted by phone. The leaders of the services were responsible for the distribution of the questionnaires. Based on information obtained from the service leaders during data collection, the population was estimated to comprise 150–200 individuals. Of these, 93 chose to participate in this quantitative study. The participants were from 16 to 30 years old (mean = 22.66, SD = 3.84); 47% of the participants were in the 21–25 year age group. Of the participants, a somewhat larger proportion were female (56%).

The majority of services (53.8%) had 2–5 participants present simultaneously, 28% had 6–10 participants present simultaneously, while smaller proportions of services had more than 11 participants (11.8%) or only one participant (6.5%). Almost all of the services had animals (96.7%).

### 2.2. Participants in the Qualitative Sample

Participants for the qualitative interviews were recruited by means of procedures similar to those used for the participants in the quantitative survey. The selected nature-based services were contacted, and service leaders were responsible for informing potential informants about this study. Participants willing to participate were registered by the service leader and put in touch with the researcher to arrange the interview.

The qualitative data comprise a total of nine participants and 20 separate interviews. Two of the participants were interviewed once, four were interviewed twice, two were interviewed three times, and one was interviewed four times. The shortest interval between the interviews was 3 months, the longest almost 1.5 years. At the time of the first interview, the participants providing qualitative data had been involved in the service for between 6 weeks and 5 years. They were between 19 and 26 years old, and the majority were female. All the interviewees had in common difficult childhoods and problems that started in lower secondary school. They also shared the experience of dropping out of upper secondary school studies. Mental health problems were a challenge for all the interviewees. Common to almost all the interviewees was the desire to be able to work fulltime in an ordinary job, and to be self-reliant. That was their common dream and goal.

### 2.3. Measures in the Quantitative Sample

A questionnaire comprising questions about the participants and about the nature-based service that they participated in was developed for this study. This questionnaire included questions about the participant’s background, previous experiences and knowledge about nature-based services, and questions about experiences from participating in these services. Questions about social relations at the services were constructed based on previous research on nature-based services, and focused on relations to the other participants, the service leader and the animals. In addition, three standardised instruments were included—The Social Provisions Scale (SPS), the Hopkins Symptoms Checklist (HSCL-10), and the Sense of Coherence Scale (SOC-13). The first two of these are reported on in this study.

#### 2.3.1. Social Provisions Scale

The original SPS consists of 24 items that measure a person’s perceived provisions of social support. It was developed by Cutrona and Russell [27] based on Weiss [22] theory on social provisions. A revised eight-item version of the scale is used in this study. For further details, see Steigen and Bergh [28]. The eight items are formulated as assertations (e.g., ‘I have relationships where my competence and skills are recognized’) and have the response format: strongly agree (4), agree (3), disagree (2), and strongly disagree (1). A high score on the SPS indicates high perceived social support [28].

#### 2.3.2. Hopkins Symptom Checklist 10

The HSCL-10 is a short version of Hopkins Symptom Checklist (HSCL-25) and measures symptoms of depression and anxiety [29,30]. The questionnaire contains 10 items describing symptoms or common problems and asks the informants to rate the extent to which these problems have bothered them in the last week (like, e.g., ‘felt that everything is a struggle’ or ‘felt constant fear and anxiety). The items have four response categories: not been affected at all (1), not been affected much (2), been affected quite a lot (3), and been affected a great deal (4). A mean value based on the 10 variable values (raw scores) can be computed. In previous studies, the cut off used is 1.85, suggesting that those with a mean score above 1.85 are considered to have symptoms of anxiety and depression [30]. In this paper, the results from the HSCL-10 are referred to as the participants’ mental health.

#### 2.3.3. Psychometric Properties

The psychometric properties of the SPS-8 and HSCL-10 were analysed using the polytomous Rasch model [31]. The property of invariance of measurement is a core characteristic of the Rasch model which allows for testing whether the items work invariantly across different groups of participants (e.g., gender) [32]. If data conform to the Rasch model, then the use of person measures based on the summation of raw scores is justified. RUMM 2030 was used for the Rasch analysis [33].

The Rasch analysis of the HSCL-10 showed satisfying psychometric properties, with a person separation index (PSI) (analogous to Cronbach’s Alpha) of 0.869 (details on the Rasch analysis conducted are available on request from the corresponding author). The Rasch analysis for SPS-8 also showed satisfying psychometric properties with a PSI of 0.692 [28]. Based on the Rasch analysis [28], the SPS-8 is only used as a composite measure in this present study—hence, the different dimensions in the instrument are not analysed nor reported.

### 2.4. Qualitative Interviews

The qualitative interviews were conducted using a semi-structured interview guide. The data were collected for use in the course of a process study that sought information about processes occurring over extended periods, which means that some of the participants were interviewed several times. The interview guide focused on participants’ own experiences with the services and the factors that had been the most important for them. Questions about expectations, the service leader and the other participants, and contact with animals and nature were included.

### 2.5. Data Analysis—Quantitative Data

Given that the data fit the model, the Rasch analysis transforms the non-linear raw scores into person values on a linear interval logit scale [34]. In the statistical analysis, these logit-transformed values from the Rasch analysis for the two scales—SPS-8 and HSCL-10—are used. The only exceptions are when mean values from the summation of raw scores are used for comparison with previous studies using raw scores, and when using a cut-off score of 1.85 for the HSCL-10, also for purposes of comparison.

### 2.6. Data Analysis—Qualitative Data

The qualitative data were analysed using qualitative content analysis, following the principles described by Graneheim and Lundman [35]. The transcribed interviews were read through several times to obtain an understanding of the overall content and to identify meaning units. Meaning units are sentences or paragraphs that represent interesting statements in relation to the overall aim of this study. Meaning units were further abbreviated (condensed) and then the condensed meaning units were interpreted and abstracted into codes and the codes were arranged and sorted into sub-categories. Following further analysis, the sub-categories were arranged into three main categories. The analysis was not a linear process and involved a constant back and forth between the whole and the parts of the text. The qualitative interviews were all analysed separately, and no process data are used in this present study, meaning that the analysis did not focus on developments from the first to the last interview. Some of the participants are interviewed several times, hence the data from some participants are richer than that from others.

### 2.7. Ethical Considerations

The Norwegian Regional Ethics Committee for Southeast Norway (2012/372) approved the quantitative survey. A letter informed the participants that participation was voluntary, that they would not be identified in the results, and that actively returning the completed questionnaire counted as consent. Four participants from two services answered the questionnaires in structured face-to-face interviews. These participants signed a written letter of consent before the interviews.

This qualitative study was approved by The Norwegian Centre for Research Data. Informed written consent was obtained from all the participants.

## 3. Results

### 3.1. Mental Health and Social Support among Participants in the Survey

Slightly more than half of the participants (54.4%) reported symptoms of mental health problems according to the HSCL-10, and the mean HSCL-10 score was 20.88 (SD = 8.46). This is slightly lower than among a clinical population (18–30 years old) in mental health care (mean = 24.81, SD = 6.60) and higher than among a general population of young adults aged 18–19 years (mean HSCL-9 = 16.70, SD = 6.02) [36].

Regarding supportive relations in the services, the majority of the participants responded strongly agree to the statement that they received support from the service leader in solving problems (81.5%), that the service leader appreciated their work (83.9%), and that the service leader was important for them to have a good time at the service (72.0%) (Table 1). Almost half of the participants responded strongly agree to the statement that they received support from the other participants in solving problems (49.4%), and the majority responded strongly agree to the statement that they experienced strong solidarity among the participants (68.2%). Just over half of the participants (54.4%) responded strongly agree to the statement that being with the other participants was important for them to have a good time in the service (Table 1). Half of the respondents responded strongly agree to the statement that the animals could give them social support at the same level as people do (50%) (Table 1).

Overall, the participants reported good perceived social support according to the SPS-8, meaning that a large proportion of them obtained scores at the upper end of the scale.

The correlation between HSCL-10 and SPS-8 was negative (r = −0.142, *p* = 0.179), indicating that when the SPS-8 score increases, the HSCL-10 score slightly decreases. Higher perceived social support therefore means better mental health on average. This correlation is, however, not statistically significant. Of the participants with symptoms of mental health problems according to HSCL-10, a larger proportion reported lower degrees of social support (62.7%) than those without symptoms of mental health problems (37.3%).

Of those strongly agreeing that they had support from the other participants in the service, a lower proportion reported symptoms of anxiety and depression (48.8%) than those not strongly agreeing that they received support from the other participants (63.4%) (not shown in a table).

### 3.2. Results of the Qualitative Analysis

Three main categories, namely acknowledgement, personal development, and safety, emerged from the analysis of the interviews. These categories are further presented with sub-categories and quotations (Table 2).

#### 3.2.1. Acknowledgement

Acknowledgement appears to be an important quality of the services, and is obtained by means of several factors, represented by the sub-categories being seen, self-determination, and adapted work.

The participants are ‘seen’ and get attention in the services. They are cared for, they feel welcomed, and experience that the service leaders are not giving up on them. Someone is always happy to see them and there is a welcoming atmosphere, as one of the participants asserted:

*“So, there is always someone who is happy to see you here. If you are going in to the goats, for example, they start jumping on you and … you know … things like that …”*

The participants are also ‘seen’ in the sense that their feelings are sensed, and sometimes mirrored, by the animals. One participant stated:

*“You can feel it on the whole horse, if you have a bad day, then yes … yes, at least noticing it on Loke (a horse) … that … if I have a bad day, then he nearly also has it a little bad somehow …”*

The services are flexible with a great degree of self-determination, and participants are included in the decisions and plans made in the services. Work tasks and activities are adapted to the participants’ needs and wishes. Many of the participants described themselves as restless, having had problems sitting still and paying attention at school. The activities in the nature-based services are varied and mostly practical and physical. At the same time, a wide spectrum of activities allows participants to choose a pace that is adapted to their day-to-day functioning. Many of the activities or work tasks can provide time for self-reflection, as one participant asserted:

*“Yes, like spreading dirt. I like it … because you are working but you do not need to think … or you can think.”*

The work tasks are also understandable and meaningful, and the participants can ‘learn by doing’. At the same time, they receive guidance from the service leaders when this is required.

#### 3.2.2. Personal Development

This category reflects the personal challenges and developments described by participants and includes the sub-categories of social insecurity, meaning something to others, strengthened social skills, and improvement.

The majority of the participants struggled with social insecurity, low self-confidence, and bad social experiences in their pasts. Some of them explicitly said that they have social anxiety, while others described behaviour that indicates such struggles. Some of them were fundamentally sceptical about other human beings and expressed insecurity regarding social roles. Many of them were very nervous before starting in the service and initially had problems with attendance. They also described it as good to be with other people in the service, and that this reduced their anxiety. As one participant indicated:

*“If I haven’t been here for two, three days or something like that … then I can feel that the nerves are coming back … because then I haven’t been among people, so that (being around people at the service) helps me a lot.”*

Participants described a sense of responsibility towards the service leader, the other participants, and the animals in the services, which made them feel that they mean something to others, that others depend on them, and that they need to contribute by doing their tasks. Hence, the participants felt important when they were with the animals, knowing that the animals needed them. It felt good to care for other living beings, as one participant said:

*“Blister (a horse) gets mad at me when I’m not here often … when I arrive in the morning he is really mad at me … and then he calms down after I have talked a bit and cuddled him.”*

The services help participants to develop and strengthening their social skills, and they obtain a better understanding of how they could act around other human beings. Participants obtain experience of being with different people and learn to work and socialise with people they do not necessarily like, as one of them stated:

*“… and then you learn a bit, also, that you … sometimes, you just have to work with people you do not like so much and … that’s very important.”*

In addition, the animals provided them with opportunities to practice their social skills in a secure context. The animals also perceived the participants’ feelings, thereby making the participants more self-conscious. Many of them drew parallels between animal and human behaviour, and reflected on the social relations and social behaviour among both animals and humans:

*“… and you learn a lot if you can keep calm around the animals and understand them, kind of … and then you learn a lot … and you can also treat other people like you treat animals, you can use it towards people also ….”*

All the participants described an *improvement* in themselves as a result of participating in the nature-based services. Their mental and physical health was strengthened, and they gained a new perspective of things: positive thoughts, losing the desire to take drugs, and describing that they get well from being there. The opportunity to experience mastery was emphasised as being important. One of the participants stated that it was the first time she had felt this:

*“Yes … that is so important … I did not believe that it was so important to experience that one can master something because, ehh … I don’t think I have felt that feeling before, to manage to master something…”*

#### 3.2.3. Safety

The analysis revealed that many of the narratives were related to an overall feeling of safety, which consists of the sub-categories of familiarity, sanctuary, safe community, and understanding.

Many of the participants had previous experiences and good feelings related to animals and farm activities. This made the setting and the activities familiar, even if the services were unknown to the participants.

The services also seem to serve as sanctuaries, where there is peace and tranquillity, and participants can get away from painful things in their everyday lives. In addition, the low pace at the services, with low perceived stress, were appreciated. The animals and nature allowed the participants to relax.

*“… it’s like far away from the city, it’s good, nature … it’s like all the painful things are a hundred miles away from here, and that’s really nice.”*

A welcoming and inclusive atmosphere is an important quality: nature, animals, and humans are all contributing to this, making the service a safe community for the participants. This was especially highlighted by those of the participants who were afraid or anxious before they started in the service, because they had feared starting there.

Substantial collaboration, common activities, and common meals characterise the services. Nevertheless, there are also possibilities for withdrawal if participants need time on their own. Working together created close relationships between the participants, as well as between the participants and the service leaders. Participants described solidarity between the participants, and that it was good to be together. The animals also provided company, and even the one participant who did not want to work with the animals acknowledged that it would have been miserable without the animals present.

Animals have a body language that is easy to read, which means that the participants could easily grasp what they communicated. The participants who had felt unsecure when communicating with other human beings and had been sceptical about humans because of their experiences with lies and dishonesty in the past appreciated this. Honesty was essential for many of the participants to feel safe.

*“… animals don’t judge you … they don’t look at you in an ugly way… right … things like that … it’s not things that I think about … it is just … you just notice it … and they are so cosy … and you can sit and cuddle with them and … strange and sweet things … and so like just being together with them …”*

That the services offered possibilities for those who were having bad days and feeling exhausted made the participants feel *understood*, which in turn motivated them to push themselves, as one said:

*“… it’s not embarrassing to say I do not have the energy to work more … they don’t get mad … and that also makes it a little safer to go on … it does.”*

One participant highlighted the importance of the fact that the leaders have the duty of confidentiality, and that it was good to know that they had experience with people having mental health- or drug-related problems. Peer support is also an important quality; it is safe and easy to talk together when participants share common experiences and can help and support each other.

## 4. Discussion

The aim of this paper was to uncover different dimensions of social support in relation to mental health among young adults with mental health problems participating in nature-based services, using both qualitative and quantitative data.

The results illustrate that the participants experience emotional support by feeling cared about and being seen in many aspects when they are in the services. Animals provide the opportunity for intimacy and comfort and respond to participants’ feelings. Previous research has also reported that participants feel understood by the animals, that they do not judge, that they provide closeness and warmth and make participants feel safe [4,13]. In addition, through their actions, the service leaders show that they care about the participants. Previous research has pointed to the importance of the emotional support provided by service leaders [37]. Emotional support hence seems to be present in all the main categories of the qualitative analysis.

Participants also seem to receive esteem support in the services. They describe positive personal development resulting from participating in the services, and they indicate that their social security is strengthened, as illustrated, for instance, in the main category of personal development. The quantitative results indicate that there is a positive relationship between social support and mental health, implying that when one of these factors is strengthened, the other also tend to increase. Even though clear causal conclusions cannot be drawn from this material, it can be hypothesised that when participants receive esteem support in the services, this leads to a stronger perceived social support and, potentially, as a consequence, fewer symptoms of mental health problems. Results from previous studies support the findings that nature-based services facilitate personal development [6]. Service leaders also provide esteem support by facilitating participants’ experiences of mastery, supported by the main category of acknowledgement. This kind of esteem support provided by the farmer is also described by Ellingsen-Dalskau [38]. In addition, the results support the view that participants receive esteem support, with a majority of the participants strongly agreeing that the service leader appreciates their work. Positive feedback and appreciation of their work by service leaders have also been found to be important for participants in previous studies of nature-based services [37].

The quantitative results indicate that the leaders help the participants with problem-solving and information and that the participants help one another, indicating that they receive informational and instrumental support in the services. As illustrated by the main category of acknowledgement, participants’ experiences of being able to turn to the service leader for help solving practical tasks and other problems are valued. That participants receive both informational and instrumental support in nature-based services has also been found in other studies [37,38].

The services also seem to facilitate social integration, as seen by the qualitative category safety. Strong solidarity among the participants is described, and the other participants and the service leader are important for the participants to have a good time at the service. The participants describe a feeling of being part of a group that shares common interests and concerns. The large amount of common activities that involve the participants and the leader strengthen the feeling of safety and affiliation. The same phenomena have been observed in earlier research [3,7,10]. Sharing concerns and supporting one another seemingly contribute to this dimension of social support.

In the main category of personal development, there are descriptions relating the possibility of providing care to other living beings, fellow participants as well as animals. Nearly all the services have animals present. Previous research supports the idea that taking care of animals is seen as meaningful [4,7].

The SPS-8 comprises the four dimensions of emotional, esteem, informational, and instrumental support [21]. Participants scored high on the SPS-8, which indicates a high degree of perceived social support. From the qualitative material, we can highlight factors that probably contribute to those high scores. Two dimensions of social support are not included in SPS-8, namely social integration and opportunities for nurturance. Even though they are not included in our quantitative study, these dimensions—based on the qualitative material—emerge as important for participants in nature-based services.

The term social is primarily used to describe interaction between humans. However, the results indicate that animals may fulfil social needs for humans. In this paper, support from animals is therefore treated as equal to support from humans and is discussed as part of the social support concept.

As discussed above, the results suggest that animals may contribute with emotional support and provide opportunities for nurturance for the participants. This also indicates that the animals may have a specific role in the establishment and development of trust, as a first stage on the path to increased trust, which includes trust in human beings. The qualitative results support this hypothesis as participants experience that animals do not condemn them and that being with animals feels safe. Emotional support and the opportunities for nurturance provided by the animals may be of specific importance to the participants with more pronounced mental health problems. Animals’ potential importance for specific participant groups in nature-based services requires further research.

The participants in the qualitative part of this study describe improvements and relate this to better mental and physical health, positive thoughts, and decreasing desire to use drugs—in addition to the overall experience that their health has improved because of their participation in the services. This indicates that the basic qualities of the nature-based services play an active part in strengthening social support and mental health.

The results point to the importance of social support in nature-based services and elaborate on the dimensions and meaning of social support. A basis for further research on social support in these services has been created. Furthermore, the results may encourage different services in mental health and social work practices—nature-based and others—to be more attentive to the meaning of social support in their services.

A limitation of this study is the small number of participants from whom quantitative data were obtained. Nevertheless, the data collection process and the mapping of services was thorough, and it is likely that all potential informants were offered the opportunity to participate. A limitation might also be the gender skewness in the qualitative sample, with the majority being female, while in the quantitative sample the gender distribution is more even. However, also in the quantitative sample, most of the participants are female. A strength of this study is its methodological uniqueness in the field. Quantitative data on nature-based services, both in Norway and internationally, are limited and, to our knowledge, no previous study has used a combination of quantitative and qualitative methods in attempting to describe the dimensions of social support in relation to mental health among young adults with mental health problems participating in nature-based services. Therefore, this study contributes important information useful in developing health promotion programs in similar client/patient groups.

## 5. Conclusions

The results indicate that various dimensions of social support are present in nature-based services and that they may contribute to strengthening participants perceived social support and mental health in various ways. This hypothesis, however, requires further research as causal conclusions cannot be drawn from these data. Nevertheless, nature-based services provide important dimensions of social support to the participants. In particular, the results demonstrate the presence of emotional and esteem support in the services. The services seem to help participants feel safe and at home, as well as teaching them how to establish contact with people [4,10]. The results demonstrate that social vulnerability is a major concern for the participants and establishing social safety and competence may therefore be the most important task for these services. The animals may serve as an important factor, in learning how to build up trust, and by providing emotional support to participants. Nature-based services may be a helpful intervention for young adults with mental health problems. The unique context of these services, including nature and animals, adds special qualities to mental health and social work practices.

## Figures and Tables

**Table 1:** Social relations at the nature-based services.

Social Relations at the Service	Strongly Agree % (N)	Partly Agree % (N)	Partly Disagree % (N)	Strongly Disagree % (N)
I get support from the farmer/leader at the service to solve problems	81.5%	17.4%	1.1%	
	(75)	(16)	(1)	-
The farmer/leader at the service show that they appreciate my work	83.9%	15.1%	1.1%	
	(78)	(14)	(1)	-
The service leader(s) are important for me to have a good time at the service	72.0%	24.7%	3.2%	
	(67)	(23)	(3)	-
It is good solidarity among the participants	68.2%	28.4%	2.3%	1.1%
	(60)	(25)	(2)	(1)
I get support from the other participants to solve problems	49.4%	31.0%	11.5%	8.0%
	(43)	(27)	(10)	(7)
Being with the other participants is important for me to have a good time at the service	54.5%	37.5%	5.7%	2.3%
	(48)	(33)	(5)	(2)
I feel that the animals can give me social support on the same level as other people	50%	34.1%	8%	8%
	(44)	(30)	(7)	(7)

**Table 2:** Results of the qualitative analysis, main categories with sub-categories.

Acknowledgement	Personal Development	Safety
being seen self-determination adapted work	social insecurity meaning something to others strengthened social skills improvement	familiarity sanctuary safe community understanding

## Data Availability

Data are held securely by the research team and may be available upon reasonable request and with relevant approvals in place.
